# A Synthetic Community System for Probing Microbial Interactions Driven by Exometabolites

**DOI:** 10.1128/mSystems.00129-17

**Published:** 2017-11-14

**Authors:** John L. Chodkowski, Ashley Shade

**Affiliations:** a Department of Microbiology and Molecular Genetics, Michigan State University, East Lansing, Michigan, USA; b Department of Plant, Soil and Microbial Sciences, Program in Ecology, Evolutionary Biology, and Behavior, The DOE Great Lakes Bioenergy Center, and The Plant Resilience Institute, Michigan State University, East Lansing, Michigan, USA; University of California, San Diego

**Keywords:** *Burkholderia*, *Chromobacterium*, *Pseudomonas syringae*, community ecology, microbial metabolomics, model microbial systems, synthetic communities

## Abstract

Though most microorganisms live within a community, we have modest knowledge about microbial interactions and their implications for community properties and ecosystem functions. To advance understanding of microbial interactions, we describe a straightforward synthetic community system that can be used to interrogate exometabolite interactions among microorganisms. The filter plate system (also known as the Transwell system) physically separates microbial populations, but allows for chemical interactions via a shared medium reservoir. Exometabolites, including small molecules, extracellular enzymes, and antibiotics, are assayed from the reservoir using sensitive mass spectrometry. Community member outcomes, such as growth, productivity, and gene regulation, can be determined using flow cytometry, biomass measurements, and transcript analyses, respectively. The synthetic community design allows for determination of the consequences of microbiome diversity for emergent community properties and for functional changes over time or after perturbation. Because it is versatile, scalable, and accessible, this synthetic community system has the potential to practically advance knowledge of microbial interactions that occur within both natural and artificial communities.

**IMPORTANCE** Understanding microbial interactions is a fundamental objective in microbiology and ecology. The synthetic community system described here can set into motion a range of research to investigate how the diversity of a microbiome and interactions among its members impact its function, where function can be measured as exometabolites. The system allows for community exometabolite profiling to be coupled with genome mining, transcript analysis, and measurements of member productivity and population size. It can also facilitate discovery of natural products that are only produced within microbial consortia. Thus, this synthetic community system has utility to address fundamental questions about a diversity of possible microbial interactions that occur in both natural and engineered ecosystems.

**Author Video**: An author video summary of this article is available.

## Abstract (precis)

Understanding microbial interactions is a fundamental objective in microbiology and ecology. The synthetic community system described here can set into motion a range of research to investigate how the diversity of a microbiome and interactions among its members impact its function, where function can be measured as exometabolites. The system allows for community exometabolite profiling to be coupled with genome mining, transcript analysis, and measurements of member productivity and population size. It can also facilitate discovery of natural products that are only produced within microbial consortia. Thus, this synthetic community system has utility to address fundamental questions about a diversity of possible microbial interactions that occur in both natural and engineered ecosystems.

## INTRODUCTION

There is modest knowledge about how microorganisms interact with each other in their native habitats and whether these microbial interactions have implications for emergent community or ecosystem properties. Microorganisms can communicate chemically, and these chemical interactions underlay a range of relationships from commensalism to antagonism (1–3). Because of the specificity of many microbe-microbe relationships, it is thought that most microorganisms produce certain chemical products only within a particular consortium (1, 4–7). However, understanding of relatively well-described microbial interactions often is incomplete. For example, sensitive mass spectrometry was employed to discover new components of an interaction between *Bacillus subtilis* and *Streptomyces coelicolor* (7), which suggested that knowledge of this interaction was limited despite having been studied previously. Investigations of microbial exometabolite production have been predominantly focused on the analysis of a single taxon or pairs (8–10) of microbial taxa rather than on multimember profiling (11). However, the collective abilities of microbiomes to produce and exploit extracellular enzymes have been hypothesized to be key in discriminating situations in which microbial community structure has implications for ecosystem processes like carbon and nitrogen cycling (12, 13). These studies and others suggest that most microbial interactions remain obscure and that improved understanding of some of these interactions likely will provide important insights into microbial community functions.

Synthetic microbial systems recently have garnered reinvigorated interest because of their potential to address fundamental unknowns in microbial ecology, engineering, and systems and synthetic biology (4, 7, 14). Synthetic microbial systems are a key approach used in microbial ecology to understand how microbial interactions lead to emergent properties of communities, such as resistance and resilience (7). For example, synthetic communities have been assembled from marine waters onto artificial particles to observe community primary succession and the resulting functional changes in model heterotrophic particles (15) and phototrophic biofilms (16), spatially constrained synthetic communities have been used to investigate reciprocal syntrophy (17), and computationally modeled synthetic communities have been applied to predict coculture growth given the metabolic needs of the members (18). Other recent work used a combination of metabolic flux analysis and multimember coculture to determine that the net outcome of complex interactions between an antagonist and a syntroph was not necessarily the sum of all expected pairwise outcomes, especially given particular spatial arrangements of the members (19). Other synthetic microbial systems are engineered to control and manipulate genetic circuitry toward required functions (20). These studies and others demonstrate that synthetic microbial communities can be applied in diverse and creative ways to provide insights into the dynamic biological and ecological interactions of microbiomes (21, 22), with the anticipation that these insights then can be applied to manage these communities toward desired outcomes.

We have developed a simple synthetic community system to interrogate exometabolite interactions among microbial community members. This synthetic community system permits direct investigation of chemical interactions among microorganisms via secondary metabolites, signaling molecules, and other exometabolites and allows for observation of behaviors that only occur when those microorganisms exist as part of a particular consortium. This system combines concepts and tools from systems biology, microbiology, biochemistry, genomics, and ecology and can provide both top-down and bottom-up approaches to investigate key questions in synthetic microbial ecology (7). Thus, it can advance understanding of microbial interactions within diverse natural and artificial microbiomes. It can also facilitate discovery of novel microbial products that are made given certain community memberships.

## RESULTS

### Description of the synthetic community experimental system.

The apparatus of the experimental system is a sterile microtiter plate. In the plate, each well has a 0.22-μm-pore filter bottom and the plate fits into a shared medium reservoir. The pore size of the filters physically separates each member from its neighbors but permits resource and metabolite sharing through the reservoir. This allows for observation of outcomes of chemical interactions between members. It is ready fabricated and commonly used for eukaryotic tissue culture. Any comparable product could be used for the synthetic community; we have used plates from Millipore (Darmstadt, Germany). Isolates from the habitat of interest are arrayed randomly into the plate, with a single member occupying each well at a known initial density or population size. The total number of wells occupied by an isolate can be used to calculate its proportional contribution to the total community. The plate, with its combination and arrangement of isolates, represents the level of the experimental unit and is replicated. The plates are incubated with gentle shaking to homogenize member access to media and exometabolites and to omit spatial effects (19). Because the filter bottom of one well is removed and used for transfer of media to the reservoir, as many as 95 unique members can be included in one consortium.

### Observation of known microbial interactions in the system.

**FIG 1:**
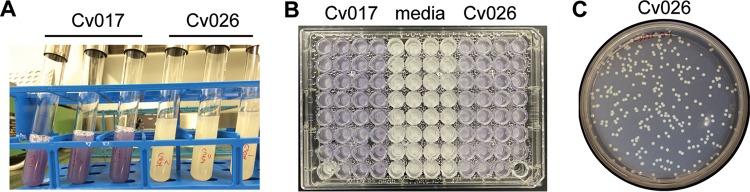
The filter plate system reproduces known microbial interactions facilitated by exometabolites. (A) Control. The *Chromobacterium violaceum* mutant strain Cv026 cannot produce acyl-homoserine lactones (AHLs), while strain Cv017 can. AHLs trigger production of the purple pigment violacein. (B) AHLs from Cv017 diffused through wells to induce quorum sensing and violacein production in Cv026. (C) Colonies of Cv026, diluted from the Transwells in panel B and plated, reverted color in the absence of exogenous AHLs.

We demonstrate that relevant microbial molecules can pass through the filter membranes into the shared community reservoir. We asked if molecules made by bacteria arrayed into the plate could be produced in biologically relevant concentrations to impact other members. We paired *Chromobacterium violaceum* Cv017, a strain that produces acyl-homoserine lactone (AHL), with the AHL biosensor *C. violaceum* Cv026. Cv026 is a strain that lacks the ability to make AHLs but produces the purple pigment violacein when exogenous AHLs are sensed (23). The violacein gene cluster is regulated by quorum sensing. Controls showed that Cv026 did not produce violacein when grown alone, as no AHLs were produced to induce quorum sensing (Fig. 1A). In triplicate, we arrayed each strain at opposite ends of a filter plate, with several wells of uninoculated medium separating them. This was done to ensure that there was not spatial heterogeneity in molecule production or sensing, which was not expected given that the plates were incubated with gentle shaking. Cv026 produced violacein when arrayed in the filter plate system with Cv017, demonstrating that it could sense the AHLs produced by Cv017 (Fig. 1B). After Cv026 produced violacein in the filter plates, wells containing Cv026 were serially diluted onto agar plates. All Cv026 colonies reverted to beige on the plate (Fig. 1C). This showed that AHLs produced from Cv017 were necessary to induce violacein production in Cv026 in the filter plates and that there was no contamination of Cv017 or relevant mutations in Cv026. We confirmed this result by comparing endpoint reverse transcription (RT)-PCR of gene expression of *vioC* to that of the housekeeping gene *rpoB*. We compared gene expression in the filter plate coculture with the test tube monoculture for Cv026 and Cv017 (see Fig. S1 in the supplemental material). This experiment showed that the synthetic community system reproduces microbial production and sensing of small molecules relevant for known microbial interactions.

10.1128/mSystems.00129-17.1 FIG S1  Endpoint RT-PCR on *vioC* and *rpoB* genes. (A) When arrayed with Cv017 in the filter plate system, Cv026 had relatively higher yield of *vioC* transcripts (replicates [Reps] 1 to 3), providing evidence that AHLs produced by Cv017 accumulated in the medium reservoir at concentrations required for Cv026 to upregulate its violacein gene cluster. (B) As a control, the constitutively expressed housekeeping gene *rpoB* had comparable transcript levels across all conditions. Download FIG S1, PDF file, 0.4 MB. Copyright © 2017 Chodkowski and Shade. 2017 Chodkowski and Shade This content is distributed under the terms of the Creative Commons Attribution 4.0 International license.

### System measurements.

Both community and member-specific parameters of the system can be measured to interpret the community outcomes. At the community level, the primary data collected are untargeted exometabolite profiles of molecules extracted from the shared medium reservoir. These profiles serve as readout of direct functional output and are relevant for member interactions. The exometabolite extraction protocol will depend on the molecules of interest (e.g., signaling molecules, small peptides, extracellular enzymes, antibiotics, etc.). Alternatively, a “global” approach could be used with multiple extraction solvents and mass spectral conditions to capture a breadth of molecules. Ecological and functional community properties can be quantified as appropriate for the scientific question.

Member-specific data can also be collected from the synthetic system. As a proxy for member success in the system, growth and viability can be determined using either live/dead staining with flow cytometry or dilution to extinction of plated well contents. As a measurement of member production, biomass can be assessed. At the end of the experiment, planktonic cells can be vacuumed onto the filters, and then the collection of filters for each member can be excised, dried, and weighed with a microbalance. Alternatively, total protein accumulation could be assayed per well. Growth and biomass are quantified relative to control conditions within the experimental design. To couple regulation with functional output, member transcript sequencing can be performed to explore linkages between gene regulation and exometabolite production. Transcript data also can inform the upregulation of cryptic pathways or help to identify exometabolites from the untargeted analysis. Biomass for each member can be removed from wells by pipetting and then combined and flash-frozen for RNA extraction. Optimally, member genome sequences would be available to be used as references for transcript assembly and analysis.

### Demonstration.

**FIG 2:**
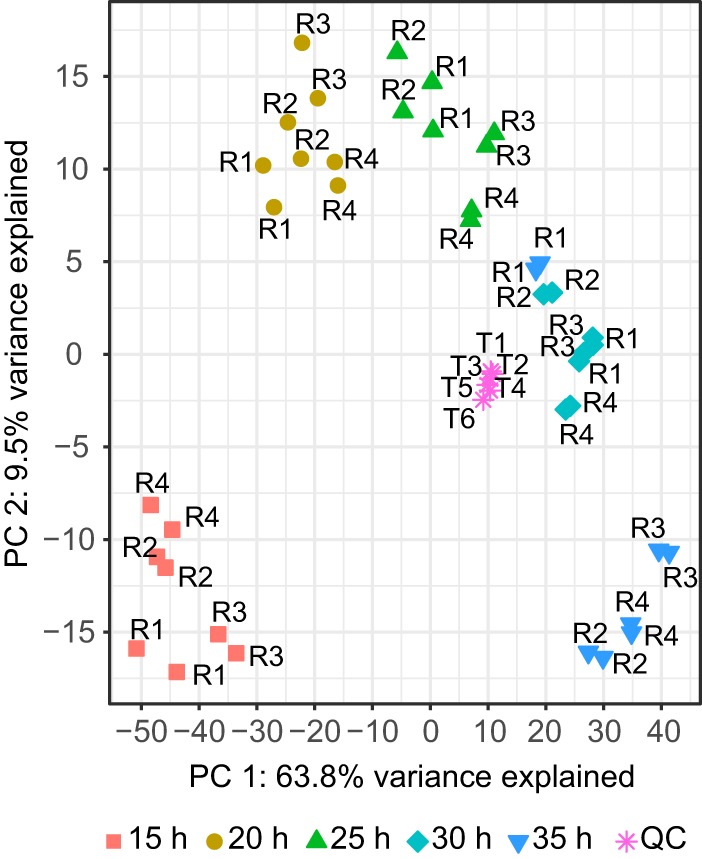
Global exometabolite changes in a three-member community compared across time and with mass spectral replication. Shown are results from principal-component analysis (PCA) of normalized, log_2_-transformed mass spectral profiles. Profiles are colored by time and labeled by replicate (R1 to R4). Quality control series (QC), a composite of all experimental samples, are labeled by analysis order (T1 to T6). A total of 977 mass features were included after quality filtering.

We demonstrated the use of the synthetic community system with a three-member community comprised of common environmental strains: *Burkholderia thailendensis* E264, *C. violaceum* SC11,368, and *Pseudomonas syringae* DC3000 (Table 1). The members were randomly arrayed in a filter plate and each occupied 31 wells so that the community was even. Over 15 to 35 h in stationary phase, we extracted shared community metabolites from the medium reservoir every 5 h and performed liquid-liquid extraction to separate the nonpolar and polar phases. Nonpolar metabolites were analyzed by ultraperformance liquid chromatography-mass spectrometry (UPLC-MS). Peak picking and retention time alignment from the UPLC-MS data set was performed in XCMS (24), and additional quality filtering steps were performed in mzMatch (25). After quality filtering, there were 977 features in the nonpolar profile. Mass spectral replicates were reproducible (Fig. 2; median Pearson’s *r* = 0.98; range, 0.96 to 0.99; all *P* values of <0.0001). As expected, our quality control (QC) samples had similar profiles even though they were analyzed at different times over the mass spectral operation (see Fig. S2 in the supplemental material; median coefficient of variation [CV], 2.62%; range, 0.45 to 12.10%). QC samples represented an average of all other profiles (Fig. 2). These data show that the synthetic community system offers experimental consistency in mass spectral results.

10.1128/mSystems.00129-17.2 FIG S2  Assessment of mass spectrometer stability using the quality control (QC) series. Principal-component analysis axis 1 scores (from Fig. 2) are plotted against analysis order and colored by sample type (experimental or QC). QC samples were an even composite of all experimental samples, run at regular intervals on the mass spectrometer to assess instrument stability and feature consistency. QC samples do not vary along axis 1, despite the fact that they are not analyzed consecutively, demonstrating that the instrument was stable over the analyses that generated this data set (54). Download FIG S2, PDF file, 0.1 MB. Copyright © 2017 Chodkowski and Shade. 2017 Chodkowski and Shade This content is distributed under the terms of the Creative Commons Attribution 4.0 International license.

**TABLE 1:** Strains used in this study

Strain	Genotype	Reference
SC11,378	Wild-type *Chromobacterium violaceum* strain ATCC 31532	55
Cv017	Sm^r^ mini-Tn*5* Hg^r^; derivative of SC11,378	56
Cv026	Sm^r^ mini-Tn*5* Hg^r^ *cviI*::Tn*5 xylE* Km^r^; derivative of Cv017	23
E264	Wild-type *Burkholderia thailandensis* strain ATCC 700388	57
DC3000	Wild-type *Pseudomonas syringae* strain ATCC BAA-871	58

We observed directional changes in the three-member community’s nonpolar exometabolite profile over time, explained by axis 1 in the principal-component analysis (PCA) (Fig. 2). There was high reproducibility in the metabolite profile changes with time across the four independent time series (PROTEST [26]; all pairwise *r*^2^ values of ≥0.938, all *P* values of ≤0.025). Thus, our results show that replicate time series were synchronous. There also were clear differences in metabolite profiles with time, as each time point had distinct profiles (global Adonis *r*^2^ = 0.758, *P* ≤ 0.01, all pairwise false discovery rate [FDR]-adjusted *P* values of ≤0.05). An exception was the 30- and 35-h profiles, which were not statistically distinct (FDR-adjusted *P* = 0.41). These results generally show that the system is robust and can facilitate observations of biologically induced changes in community exometabolites.

**FIG 3:**
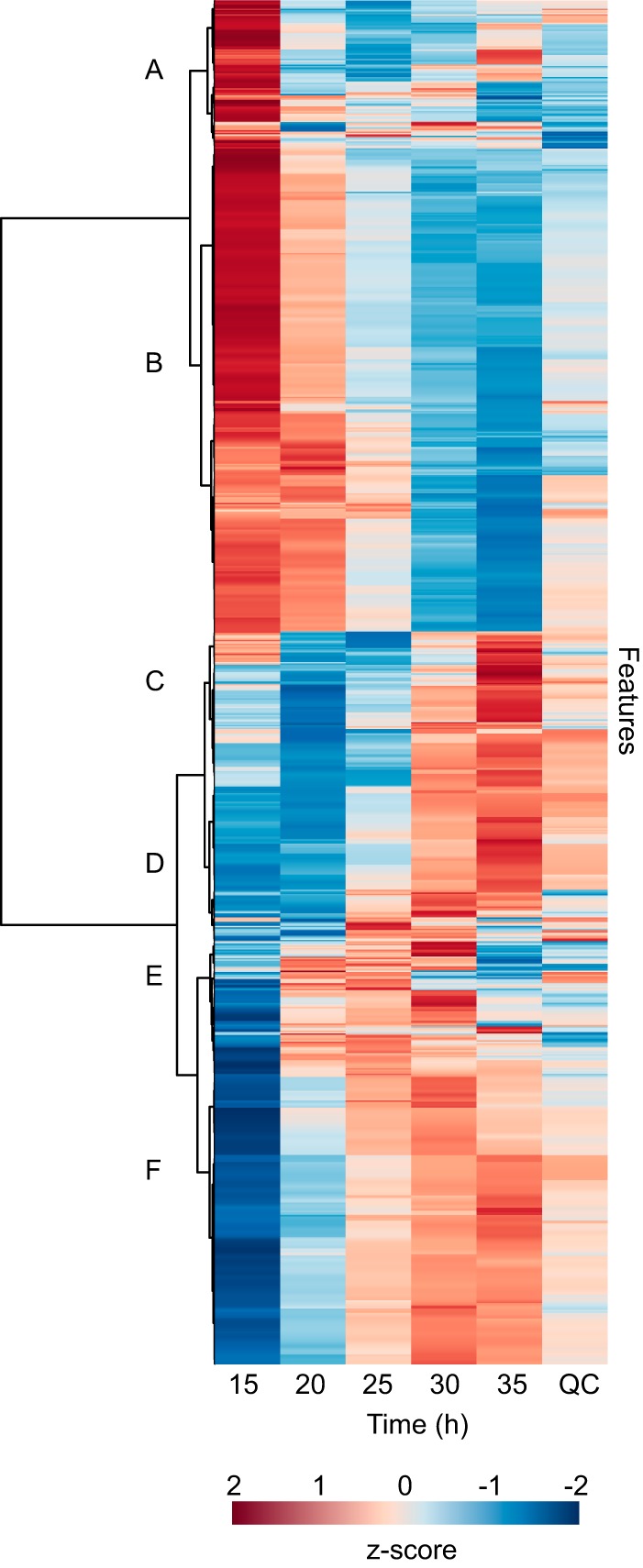
Exometabolites exhibit directional changes over stationary phase in a three-member synthetic microbial community. Shown is a heat map of 977 mass feature changes over time within a three-member community, where samples are columns and features are rows. Each sample is the average of four time point replicates, each started independently from new cultures. Euclidean distance was calculated from *Z*-scored mass spectral profiles. Features with similar dynamics were clustered by Ward’s method. Letter designations for clusters were added *post hoc* to aid in discussion. “QC” is quality control series, an even composite of all experimental samples that was run at regular intervals on the mass spectrometer to assess instrument stability and feature consistency.

To observe the common temporal patterns of features, a heat map was created using Ward’s clustering algorithm with Euclidean distances from *Z*-scored data (Fig. 3). We observed both decreases in existing features (clusters A and B) and production of new features over time (clusters D, E, and F). There were also some features that were enriched in early or mid-time points (i.e., in cluster C and in cluster E at 20 to 25 h, respectively). There were a few features that had variable dynamics, such as in cluster A. While it is outside the scope of this work to identify each of these features, these overarching patterns demonstrate that there are biological changes occurring in the three-member community’s exometabolite profile over time that are attributable to member production as well as medium depletion. A heat map including all replicates is provided in Fig. S3 in the supplemental material.

10.1128/mSystems.00129-17.3 FIG S3  Exometabolites exhibit directional changes over stationary phase in a three-member synthetic microbial community. Shown is a heat map of 977 mass feature changes over time within a three-member community, where samples are columns and features are rows (*Z* score range, −4.37 to 3.94). All profiles are included as samples, including two mass spectral replicates from each of four time point replicates. Each time series also included a medium control (NC). A quality control sample (QC), an even composite of all experimental samples, was run at regular intervals on the mass spectrometer to assess instrument stability and feature consistency. Euclidean distance was calculated from *Z*-scored mass spectral profiles. Features with similar dynamics were clustered using Ward’s method. Letter designations for clusters were added *post hoc* to aid in discussion. Download FIG S3, PDF file, 0.4 MB. Copyright © 2017 Chodkowski and Shade. 2017 Chodkowski and Shade This content is distributed under the terms of the Creative Commons Attribution 4.0 International license.

To further validate that the synthetic community system can produce the expected results in biologically complex situations, we also asked whether we could observe the expected dynamics of a known molecule within the system. We hypothesized that bactobolin, a characterized bacteriostatic molecule produced by *B. thailandensis* E264 (27), would accumulate in the medium reservoir of the three-member community over stationary phase, as previously reported for other *B. thailandensis* cultivation conditions (28). We identified a feature consistent with the mass of bactobolin using polar metabolite analysis (*m*/*z* = 383.075) (see Fig. S4A in the supplemental material). Tandem MS (MS-MS) fragments of the parent ion were consistent with those reported in the mass spectral molecule database METLIN (29) for bactobolin (see Table S1 and Fig. S4B in the supplemental material), confirming the identity of this feature as bactobolin. In addition, the feature identified as bactobolin accumulated in the media of the three-member community through time (Fig. S4C).

10.1128/mSystems.00129-17.4 FIG S4  Identification and accumulation of bactobolin. (A and B) Chromatographic traces of bactobolin from MS (A) and MS-MS (B) analyses. (C) Bactobolin accumulation in the shared medium reservoir through time (*n =* 4 integrated peak areas per time point). The bottom and top of the box are the first and third quartiles, respectively, and the line inside the box is the median. The whiskers extend from their respective hinges to the largest value (top), and smallest value (bottom) was no further away than 1.5× the interquartile range. Download FIG S4, PDF file, 0.1 MB. Copyright © 2017 Chodkowski and Shade. 2017 Chodkowski and Shade This content is distributed under the terms of the Creative Commons Attribution 4.0 International license.

10.1128/mSystems.00129-17.7 TABLE S1  Fragments observed from MS-MS analysis of bactobolin. Download TABLE S1, PDF file, 0.1 MB. Copyright © 2017 Chodkowski and Shade. 2017 Chodkowski and Shade This content is distributed under the terms of the Creative Commons Attribution 4.0 International license.

**FIG 4:**
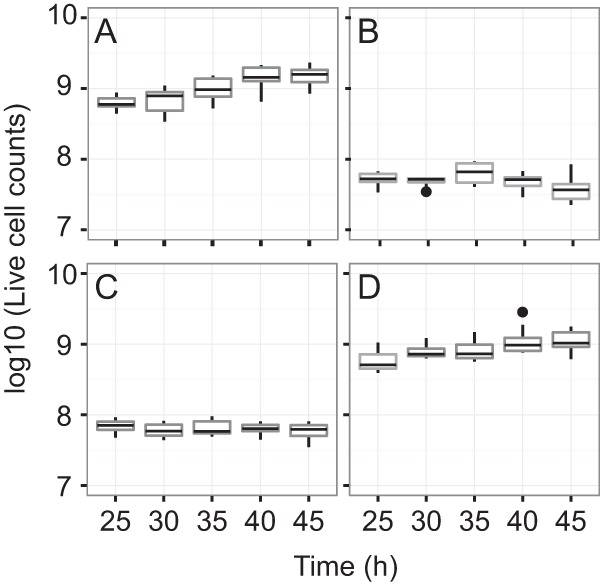
The filter plate system provides evidence of inhibition among members. Shown are changes in live cell counts of *P. syringae* over stationary phase, measured using flow cytometry of Syto9-stained cells recovered from the filter plates. Five wells per plate and two replicate plates per time point were used to assess *P. syringae* cell counts when grown in monoculture (A), the three-member community (B), coculture with *B. thailandensis* (C), and coculture with *C. violaceum* (D). Reduced counts in coculture and the three-member community (compared to counts in monoculture or with *C. violaceum*) suggest an antagonistic interaction with *B. thailandensis*.

We also demonstrated member-specific measurements from the system. We measured cells recovered over time in a three-member experiment and also from monoculture and from coculture experiments conducted in filter plates. Cell count data using live/dead staining with flow cytometry had high reproducibility (median CV, 1.55%; range, 0.87 to 2.35%). These data revealed potential antagonism between *P. syringae* and *B. thailandensis*, as evidenced by the reduced *P. syringae* live cell counts when grown in the same consortium as *B. thailandensis* compared to its cell counts in monoculture or when grown only with the third community member, *C. violaceum* (Fig. 4). To the best of our knowledge, this is the first time that antagonism between *B. thailandensis* E264 and *P. syringae* DC3000 has been observed, although previous studies have shown that *B. thailandensis* can produce antibacterials (28, 30). This is relevant because *P. syringae* spp. are common plant pathovars, and we suggest subsequent work should explore this interaction with biocontrol applications in mind.

We observed consistent population sizes for each community member over stationary phase (see Fig. S5 in the supplemental material), suggesting that, for this community, exometabolite interactions that occurred during stationary phase impacted functional output without impacting standing population sizes. Thus, outcomes of member exometabolite interactions did not drastically alter population sizes in this consortium. In contrast, the expectation in ecological compensatory dynamics is that population sizes of competitors are negatively correlated, such that an increase in the more fit population corresponds with a decrease in the less fit population. The observation of static population sizes in the synthetic system is important because it suggests that we can identify signatures of microbial interactions that are not necessarily indicated by changes in population size. Microbial interactions without obvious growth outcomes may be more cryptic and require more precise characterization than what total cell counts can provide, but would be observable in this system if driven by exometabolites.

10.1128/mSystems.00129-17.5 FIG S5  Changes in live cell counts over stationary phase measured using flow cytometry of Syto9-stained cells that were recovered from the filter plates. Five wells per plate and two replicate plates per time point were used to assess cell counts in the three-member community. (A) *P. syringae*. (B) *C. violaceum*. (C) *B. thailandensis*. Download FIG S5, PDF file, 0.1 MB. Copyright © 2017 Chodkowski and Shade. 2017 Chodkowski and Shade This content is distributed under the terms of the Creative Commons Attribution 4.0 International license.

Taken together, our synthetic community results demonstrate that this new system can reveal global exometabolite dynamics and interactions among microbial community members.

## DISCUSSION

### Application to the fields of systems microbiology and microbial ecology.

The synthetic community system introduced here can be applied to address a variety of timely and compelling questions in systems and community microbiology. First, the synthetic system can be used to address fundamental questions about the consequences of community diversity. Membership manipulations can be performed to address the importance of community richness (total number of taxa) and structure (relative contributions of members) on member interactions and emergent community properties. For example, it has been suggested that microbial community structure matters most for function in the production of exometabolites such as enzymes and polysaccharides, which have implications for biogeochemical processes like carbon cycling (12) and N fixation (13). The system could be used to interrogate these exometabolites directly. Similarly, the system can be used to investigate temporal changes in member interactions, to determine how member interactions change in response to perturbations, and to experimentally evolve microbial interactions within communities. These and similar lines of inquiry will allow researchers to ask how microbial interactions and products change as the result of controlled and specific environmental cues. Finally, the system can facilitate discovery of natural products. Member genomes can be mined for cryptic metabolic pathways from bioinformatic predictions, and this information could then be coupled to synthetic community manipulations to observe regulation. Novel or unknown exometabolites are likely to be discovered in an untargeted analysis of the community exometabolites, and their chemical structures and activities can be pursued subsequently.

### Advantages and limitations.

This synthetic community system offers several experimental advantages. First, the synthetic system offers an opportunity to interrogate a relatively simple community within well-defined experimental conditions (7). It allows researchers to focus specifically on community outcomes driven by exometabolites and not by physical contact, as well as the causes and consequences of those outcomes. These interactions can be challenging to target in other mixed batch or bioreactor systems. The system also is versatile because it can be used with microbial consortia from any ecosystem and adjusted to simulate environmental conditions of interest. The system also is scalable, not only for increasing the overall community diversity, but also for moving toward higher-throughput and high-content screens for molecules and community outcomes of interest. Finally, the system is accessible. The filter plates are available to any lab, and manual manipulation of the system without specialized equipment is feasible. We suggest that the most limiting factor is access to mass spectrometers and expertise in mass spectral analysis, which if not available locally is accessible via research support facilities.

As is true for any laboratory-scale experimental system, this synthetic community system also has several limitations. First, all possible types of microbial interactions are not observable using this system. Some exceptions include interactions that are contact dependent or in which chemical exchanges and physical contact are not clearly distinguishable or independent. The system instead allows for control of the influence of physical spatial structure on microbial interactions, which has been shown to be important for stabilizing some communities, especially for highly structured environments like biofilms or soil matrices (e.g., 17, 19, 31, 32). However, because members are spatially partitioned yet permitted to interact chemically, this system allows the researcher to control for spatial effects without a typical limitation of homogenous coculture: overgrowth of one member that prevents long-term observations of community interactions (17).

Not all relevant microbial exometabolites and community outcomes will be observable in this system. Specifically, exometabolites that have a rapid turnover or that are concentration dependent in ranges outside the system’s physical constraints and imposed experimental conditions will be inaccessible (e.g., total medium volume or experiment duration, respectively). The ability to observe a molecule and its community outcomes also depends on the sensitivity of the microbial sensing/signaling systems involved, which will depend on the members’ capabilities. Also, this system may not be ideal for situations in which the local accumulation of an exometabolite inhibits the reaction generating the exometabolite, but this will depend on the duration of the experiment relative to the expected rate of exometabolite accumulation in the reservoir. Finally, interactions that are reliant on volatiles that may off-gas during plate shaking will not be observable.

Only cultivable organisms can be applied easily to the system, and so if the most functionally important or prevalent members of a community are yet uncultivable, their interactions will be difficult to observe with this system. However, cultivation methods are improving, in part because cultivation conditions can be informed by metagenome and (meta)transcriptome data (33), and there is evidence that growing microbial community members in cohorts from the environment can improve isolate recovery (34). Thus, this synthetic community system could be used to provide insights into the precise memberships and molecules required to bring new isolates into laboratory culture.

A general limitation of any system used to observe exometabolites is that many microbial exometabolites are unknown and difficult to identify. We anticipate that this limitation will be overcome as technology and infrastructure for exometabolite identification advances. Analysis pipelines to integrate exometabolite data with other microbial omics approaches, such as transcripts and metabolic flux analysis, are also in active development (35, 36). Therefore, the first experiments using the synthetic community system will face necessary challenges in spearheading analysis and integration approaches.

There are general criticisms offered for using model or laboratory-scale systems in microbial ecology, and a common concern is that any model cannot mimic natural conditions and therefore is not biologically relevant. The synthetic community system described here is an artificial, simplified model. However, it is a model that offers many advantages specifically for understanding the chemical feedbacks on community ecology driven by microbial interactions, which is a key goal of synthetic microbial ecology (7). These interactions have the potential to occur in nature, especially when thoughtful experimental designs are employed to (i) include organisms that are naturally cooccurring or have evidence of interactions and (ii) manipulate the pertinent primary drivers of natural ecosystems. Furthermore, important advances in ecology and evolution have been made using model systems (37–40). Microbial synthetic systems especially have offered insights because of their malleable communities and molecular tools for understanding population dynamics (4, 41–43). Thus, researchers continue to use model systems because they can inform as to both biological potential and constraints in nature. When complemented with careful studies *in situ*, the synthetic community system described here can serve to discover and interrogate microbial interactions, the signatures of which may otherwise be unobservable within the complexity of natural systems.

## MATERIALS AND METHODS

### Filter plate preparation.

To prepare and use the filter plates for experiments, all protocols were performed using an aseptic technique in a biosafety level 2 cabinet. For the synthetic community experiments, we used sterile filter plates with 0.22-μm-pore polyvinylidene difluoride (PVDF) filter bottoms (Millipore MAGVS2210). These are also referred to as “Transwell plates” in the tissue culture literature. Prior to use, filter plates were washed three times with sterile water using a vacuum apparatus (NucleoVac 96 vacuum manifold; Clontech Laboratories). The filter of well H12 was removed with a sterile pipette tip and tweezer, and 31 ml of medium was added to the reservoir through well H12. Each well was then filled with 130 μl of culture or medium.

### Validation of the synthetic community system by the quorum sensing experiment.

We investigated the ability of quorum sensing molecules produced by populations arrayed in some wells to be sensed by nonproducing but receptive populations in other wells. Cv017 and Cv026 (Table 1) were inoculated in half-concentration Trypticase soy broth (TSB50) from overnight growth on half-concentration Trypticase soy agar (TSA50) in 3 replicate plates and grown at 29°C for 10 h. Cv017 was diluted to an optical density at 600 nm (OD_600_) of 0.2, and Cv026 was diluted to an OD_600_ of 0.075. Dilutions were either inoculated into test tubes (monoculture control growth) or a filter plate containing TSB50. Wells from columns 1 to 4 were inoculated with 130 μl/well Cv017 culture, columns 5 to 8 were inoculated with 130 μl/well fresh TSB50, and columns 9 to 12 were inoculated with 130 μl/well Cv026 culture. Control cultures in test tubes were incubated at 29°C at 200 rpm (model 4353; Thermo Scientific). After 16 h, cultures were flash-frozen in liquid nitrogen and stored at −80°C. Filter plates were incubated at 29°C with gentle shaking (0.32 relative centrifugal force [rcf]) for 26 h. After 26 h, 10 µl from 5 wells containing Cv026 and 10 µl from the shared medium reservoir were serially diluted (10^−4^) and plated on TSA50 for 24 h at 29°C. The remaining Cv026-innoculated wells were pooled, flash-frozen in liquid nitrogen, and stored at −80°C.

Genomic DNA (gDNA) was used as a positive control in RT-PCR experiments. An overnight culture of Cv017 was grown in TSB50 at 29°C. Genomic DNA was isolated using the EZNA bacterial DNA kit (Omega Bio-Tek, Norcross, GA). We extracted RNA from the Cv017-Cv026 filter plate coculture experiment and from both test tube controls. RNA was isolated using the EZNA bacterial RNA kit (Omega Bio-Tek) according to the manufacturer’s instructions and treated with RNase-free DNase (Qiagen). Purity of RNA was analyzed on a NanoDrop spectrophotometer using a 260/280 absorbance ratio and quantified using Qubit 2.0 (Life Technologies, Inc.).

Primers for *vioC* and *rpoB* were designed with Primer3 v.0.4.0 (44) using *C. violaceum* reference sequences from NCBI’s GenBank (see Table S2 in the supplemental material). To confirm that the amplicon products were as expected, *vioC* and *rpoB* bands were excised from an agarose gel and purified using the Wizard SV gel and PCR clean-up system (Promega). Sanger sequencing was performed on the purified bands at the Michigan State Genomics Core using the forward and reverse primers from *vioC_Cv* and *rpoB_Cv*.

10.1128/mSystems.00129-17.8 TABLE S2  *C. violaceum* primer sets designed for reverse transcription and endpoint PCR. Download TABLE S2, PDF file, 0.1 MB. Copyright © 2017 Chodkowski and Shade. 2017 Chodkowski and Shade This content is distributed under the terms of the Creative Commons Attribution 4.0 International license.

PCR was performed on extracted RNA to ensure proper DNase treatment using the *vioC_Cv* primers. Fifty nanograms of RNA from each sample was added to GoTaq Green 2× master mix (containing buffer and enzyme; Promega), 0.5 μM forward and reverse primers, and nuclease-free water in a total volume of 25 µl/reaction. Thirty nanograms of Cv017 gDNA was used as a positive control and nuclease-free water as a negative PCR control. PCR conditions were as follows: 95°C for 5 min, 95°C for 15 s, 56°C for 15 s, and 72°C for 25 s, repeated 29 times from step 2, followed by 72°C for 10 min and hold at 7.4°C. Five microliters of each PCR product was run on 1% agarose gel containing 0.5× Tris-borate with EDTA (TBE) and ethidium bromide with a 100-bp DNA ladder (New England Biolabs). Electrophoresis was run for 50 min at 100 V. Gels were visualized by UV illumination.

Reverse transcription (RT) was performed using a Thermo Fisher Scientific high-capacity cDNA RT kit for both *vioC* and *rpoB* genes according to the manufacturer’s instructions. Each RT reaction mixture contained ~1,500 ng template RNA, 0.625 μM reverse primer (Table S2), 4 mM deoxynucleoside triphosphates (dNTPs), MultiScribe murine leukemia virus (MuLV) reverse transcriptase (50 U; Applied Biosystems), 1× RT buffer, and nuclease-free water in a total volume of 20 µl/reaction. An RT negative control was prepared for each primer using only nuclease-free water instead of template. The RT thermocycler conditions were as follows: 25°C for 10 min, 37°C for 2 h, and 85°C for 5 min, followed by hold at 4°C. cDNA was stored at −80°C.

Endpoint PCR was performed on cDNA after the reverse transcription reaction for both *vioC* and *rpoB* using the *Pfu* Turbo DNA polymerase kit (Agilent Technologies). Each reaction mixture contained 1 µl of RT product, 1× *Pfu* buffer, 0.2 mM dNTP, 1× *Pfu* DNA polymerase, 0.5 μM forward and reverse primers (Table S2), and nuclease-free water in a total volume of 25 μl/reaction. Thirty nanograms of Cv017 gDNA was used as a positive control, while a nuclease-free water sample served as a negative PCR control. The RT reaction without template DNA served as an additional negative control. PCR conditions for both primer sets were as follows: 95°C for 2 min, 95°C for 30 s, 56°C for 30 s, and 72°C for 45 s, repeated 29 times from step 2, followed by 72°C for 10 min and hold at 4°C. Twenty-five microliters of each PCR product was mixed with 6× loading dye (New England Biolabs) and run on 1% agarose gel containing 0.5× Tris-borate with EDTA (TBE) and ethidium bromide with a 100-bp DNA ladder (New England Biolabs). Electrophoresis was run for 50 min at 100 V. Gels were visualized by UV illumination.

### Three-member synthetic community experiments. (i) Experimental setup.

Prior to initiating the synthetic community experiments, we characterized member growth curves to determine their compatibility in our experimental conditions. To perform the experiments, we diluted overnight cultures to concentrations that would allow the members to achieve stationary phase within 1 to 2 h of one another. Freezer stocks of *B. thailandensis*, *C. violaceum*, and *P. syringae* (Table 1) were plated on TSA50 at 28°C for 36 h. Isolated colonies were inoculated in 5 ml of M9–0.2% glucose medium and grown overnight at 28°C with gentle shaking. Overnight cultures of each strain were diluted 1:20 in fresh M9-glucose medium prior to inoculation in the filter plates (130 µl/well), and the medium reservoir was filled with 31 ml M9-glucose. Filter plates were prepared as described above. For each plate, a custom R script (RandomArray.R [see the GitHub repository]) was used to randomize community member placement in the wells so that each member occupied a total of 31 wells per plate. For each of four replicate time courses (where each time course included five points assessed every 5 h over stationary phase), five replicate filter plates were prepared for destructive sampling. Filter plates were incubated at 28°C with gentle shaking (~0.32 rcf). The first plate was destructively sampled at 15 h, and each subsequent plate was destructively sampled every 5 h thereafter until 35 h. Spent medium (~31 ml) from the shared reservoir was flash-frozen in liquid nitrogen and stored at −80 °C prior to metabolite extraction.

### (ii) Flow cytometry.

Prior to analysis, live/dead gates were established using overnight cultures grown in in M9-glucose. Fifteen microliters of log-phase cells was placed in either 135 μl Tris-buffered saline (TBS; 20 mM Tris, 0.8% NaCl [pH 7.4]) (live) or 135 μl 70% isopropanol (dead) and incubated at room temperature for 10 min. Live and dead cells were then diluted an additional 100-fold (1,000-fold total dilution) and stained with the Thermo Scientific LIVE/DEAD BacLight bacterial viability kit at final concentrations of 1.5 μM Syto9 (live stain) and 2.5 μM propidium iodide (dead stain). Two hundred microliters of stained cultures was transferred to a 96-well microtiter U-bottom microplate (Thermo Scientific). Twenty microliters of sample was analyzed on a BD Accuri C6 flow cytometer (BD Biosciences) at a fluidics rate of 66 μl/min and a threshold of 500 on an FL2 gate. The instrument contained the following optical filters: FL1-533, 30 nm; FL2-585, 40 nm; and FL3, 670-nm longpass. Data were analyzed using BD Accuri C6 software version 1.0.264.21 (BD Biosciences). Live/dead gates are provided for each member (see Fig. S6 in the supplemental material). Reproducibility in cell counts was assessed using the coefficient of variation (CV) in R.

10.1128/mSystems.00129-17.6 FIG S6  Fluorescence scatter plot of live/dead cells. Flow cytometry gates for community members show how live and dead cell events are captured predominantly on the FL1 channel and FL3 channel, respectively. Download FIG S6, PDF file, 0.2 MB. Copyright © 2017 Chodkowski and Shade. 2017 Chodkowski and Shade This content is distributed under the terms of the Creative Commons Attribution 4.0 International license.

After gates were established, we used live/dead staining of cells with flow cytometry to determine member population size from cells collected at each time point. For each member, five replicate wells containing spent culture were prepared for flow cytometry analysis. From each well, 20 µl of culture was placed in 180 µl TSB. In plate arrangements where *P. syringae* was arrayed with *B. thailandensis*, *P. syringae* culture was diluted 70-fold in TBS. In plate arrangements where *P. syringae* was arrayed in monoculture or in coculture with *C. violaceum*, *P. syringae* was diluted 900-fold in TBS. Diluted cultures were stained and analyzed as described above. Flow cytometry analyses of both *B. thailandensis* and *C. violaceum* were prepared as described above, except that *B. thailandensis* was diluted 1,300-fold and *C. violaceum* was diluted 1,540-fold before staining.

### (iii) Metabolite extraction and preparation.

To prepare samples for nonpolar mass spectral analysis, 5-ml aliquots of spent medium from each filter plate medium reservoir and four M9-glucose medium controls were thawed on ice with 5 ml of 100% methanol. After thawing, solutions were transferred to glass separatory funnels that were initially washed with Liquinox (Alconox, Inc., New York, NY), rinsed with water, dried, and then rinsed three times with acetone. Three separate 5-ml liquid-liquid extractions were performed on each sample using dichloromethane, and then the combined volume of the organic layers from each extraction (15 ml) was pooled into a 50-ml canonical tube. The organic layer was dried under nitrogen (N2 evaporator system; Glas-Col). Samples were resuspended in 1.0 ml 65%:35% (vol/vol) acetonitrile–0.1% formic acid in water.

To prepare samples for polar mass spectral analysis, 2 ml of the aqueous layer from liquid-liquid extractions was evaporated using a Savant SVC 100H centrifugal evaporator. Dried samples were resuspended in 150 µl of methanol, sonicated (Branson M1800) for 30 min, further resuspended with the addition of 350 µl of acetonitrile, and centrifuged at 20,817 × *g* for 20 min, and the supernatant was filtered through a 0.22-μm-pore PVDF membrane.

### (iv) Mass spectral analysis.

Reverse-phase chromatography and mass detection of exometabolites were performed on a Waters Xevo G2-XS QTof UPLC–MS-MS instrument. For nonpolar UPLC analysis, 10 µl was injected into a C_18_ column (BEH Shield; 2.1 by 100 mm, 1.7-μm particle size; Waters, Milford, MA) maintained at 35°C. Samples were eluted at a flow rate of 0.3 ml/min under the following gradient conditions: 99% A–1% B for 1 min, followed by 1% to 99% B in 10 min, then hold at 99% B for 3 min before returning to the initial condition for 1 min. Mobile phase A consisted of 0.1% formic acid in water (pH 2.7), and mobile phase B consisted of acetonitrile. MS conditions were set as follows: mass range acquisition, 50 to 2,000 *m*/*z*; ionization mode, electrospray ionization negative (ESI^−^) sensitivity mode; scan time, 0.5 s; collision energy, 6 eV; capillary voltage, 2.5 kV; sampling cone, 40 V; source temperature, 100°C; desolvation temperature, 350°C; cone gas flow, 50 liters/h; desolvation gas flow, 550 liters/h; and low mass (LM) resolution, 4.7. For accurate mass acquisition, a lock mass of leucine enkephalin ([M − H]^−^ = 554.2615) was used. Twenty experimental samples (4 time series replicates and 5 time points/series) and 4 M9-glucose controls (1 for each time series) were analyzed twice as mass spectral replicates. All samples, including solvent blanks, were analyzed in a random order. In addition, a composite quality control (QC) sample was made by combining 50 μl from each experimental sample, including all biological replicates but excluding medium controls (45). A QC dilution series was prepared by diluting the QC sample 2-, 4-, and 8-fold. The QC sample was used to condition the column at the beginning of the mass spectral analysis (45) and to assess instrument stability at six time points over the course of the mass spectral analysis (45). The QC dilutions were analyzed at the end of the mass spectral analysis and were used to filter out noisy features as part of the data quality control prior to statistical analysis (46). Raw Waters MS data files were converted to netCDF file format using MassLynx DataBridge software (Waters, Milford, MA).

Hydrophilic interaction liquid chromatography (HILIC) mass detection of exometabolites was performed on a Waters Xevo G2-XS quadrupole time of flight (QTOF) UPLC–MS-MS instrument. For polar UPLC analysis, 10 µl of each sample was injected into an HILIC column (Cortecs UPLC, 2.1 by 100 mm, 1.7-μm particle size; Waters, Milford, MA) maintained at 35°C. Samples were eluted at a flow rate of 0.3 ml/min under the following gradient conditions: 0% A–100% B for 1 min, 0% to 52.5% A in 13 min, hold at 52.5% A for 3 min before returning to the initial condition for 5 min. Mobile phase A consisted of 5 mM ammonium acetate in water (pH 3), and mobile phase B consisted of 95% acetonitrile–5% 5 mM ammonium acetate in water (pH 3). MS conditions were set as follows: mass range acquisition, 50 to 2,000 *m*/*z*; ionization mode, ESI^+^ sensitivity mode; scan time, 0.1 s; collision energy, 6 eV; capillary voltage, 3.0 kV; sampling cone, 35 V; source temperature, 100°C; desolvation temperature, 350°C; cone gas flow, 25 liters/h; desolvation gas flow, 600 liters/h; and LM resolution, 4.7. For accurate mass acquisition, a lock mass of leucine enkephalin ([M + H]^+^ = 556.2771) was used. Samples were analyzed in blocks of 4 time series replicates, starting with 35 h and decreasing sequentially to 15 h, followed by 4 medium control samples. A solvent blank was run between each block. Given the expectation of bactobolin accumulation through time (28), samples were analyzed in this order to ensure that there was no sample carryover in each subsequent block analyzed. The sample taken at 35 h from replicate 4 was chosen for MS-MS analysis to confirm the identification of bactobolin. For MS-MS analysis, similar UPLC conditions and MS conditions were used with the following exceptions: MS set mass, 383.1; MS-MS range acquisition, 40 to 600 *m*/*z*; LM resolution, 17.0; and collision energy ramp, 20 to 80 eV.

### (v) Peak selection, quality control, and global analysis of nonpolar mass spectral data.

mzMatch version 2.0-13 was used for metabolomics analysis (25). Fifty-seven mass spectral files were analyzed: 4 time series replicates, 5 time points/series, 2 mass spectral replicates/time point, 4 negative controls with 2 mass spectral replicates each, 6 QC samples, and 3 QC dilution series samples. First, XCMS version 1.48.0 was used within mzMatch for peak selection and retention time correction (24). Then, peak grouping, peak filling, and peak filtering steps were performed in mzMatch. Filtering steps included noise filtering, medium control removal, removal of features below and above the elution gradient (<0.5 min and >18 min of retention), and QC dilution series assessment. Pearson’s correlation was used to assess congruence across dilutions for each feature and to determine if the experimental series for an undiluted feature was highly correlated with that of its 1:2, 1:4, and 1:8 dilutions (46). Features with a dilution series Pearson’s *r* of <0.9 and/or *P* value of >0.05 had an irreproducible dilution trend and were removed (46). The resulting feature by sample matrix was converted from a .peakml file to a text file and was imported into R. The coefficient of variation (CV) was calculated in R to assess reproducibility of QC samples. Duplicate retention times were removed, features with a positive Pearson correlation coefficient from the QC dilution series were removed, and features with the greatest average abundances in the QC samples were removed. The three QC dilution series samples and medium control samples were omitted from the statistical analysis of differential patterns. Missing values were changed to half the minimum value, and labels were added to comply with the MetaboAnalyst data format (47).

The feature table was uploaded into MetaboAnalyst 3.0 (47). Raw feature intensity values from the quality-filtered data set were normalized using probabilistic quotient normalization (48) and log_2_ transformed (49). The resulting mass feature table was used for statistical analysis and visualization. We used principal-component analysis to explore global changes in metabolite profiles across samples (Fig. 2). We tested for significant differences among time points using permutational multivariate analysis of variance (PERMANOVA) (50), implemented with the Adonis function in the vegan package (51). We first performed a global test with Adonis to determine as if there are any differences among any time points. If the global test was significant, *post hoc* pairwise tests between all time points were performed to determine which were different from each other (e.g., time 15 versus time 20). To correct for multiple comparisons, we used a false-discovery rate (FDR) adjustment to the *P* values for *post hoc* tests, using the p.adjust script from the base package in R (52). We used a Procrustes superimposition analysis to determine if replicate time series were coherent (26). This test was implemented with the PROTEST function in the vegan package (51). We used MetaboAnalyst to create a heat map to visualize feature changes over time. For the heat map, time point replicates were standardized with *Z* scores, and then features were averaged within a time point. A full heat map with each replicate as a separate column is provided in the supplemental material (Fig. S3).

### (vi) Bactobolin identification from polar mass spectral data.

For MS analysis targeting bactobolin, polar mass spectral data from the HILIC analysis were used. Targeted peaks were detected in MZmine 2.17 with the following parameters: intensity tolerance, 10%; noise level, 50; *m*/*z* tolerance, 30 ppm; and retention time tolerance, 0.4 min. After peak detection, peak extension was used with the following parameters: *m*/*z* tolerance, 30 ppm; and minimum height, 50. Selected peaks were joined into one data file using the join aligner function in MZmine, and peak areas were exported to a CSV file. The R package ggplot2 (53) was used to make a box plot tracking bactobolin accumulation through time.

For MS-MS analysis, the netCDF file was uploaded into MZmine to observe MS-MS fragments. Scan number 2405 was chosen to produce the fragment list provided in Table S1 because this MS-MS scan had the least parts per million errors for all fragments. Extracted ion chromatographic traces of bactobolin from the MS and MS-MS data files were generated in XCMS.

### Availability of data.

Computing workflows (source code and input files) are available on GitHub (https://github.com/ShadeLab/PAPER_Chodkowski_mSystems_2017). Mass spectral data have been submitted to MetaboLights (http://www.ebi.ac.uk/metabolights/MTBLS525).
