# Supplementary figures and images for: A Synthetic Community System for Probing Microbial Interactions Driven by Exometabolites
Source: mSystems. 2017 Nov 14;2(6):e00129-17. doi: 10.1128/mSystems.00129-17 (PMC5686522; doi:10.1128/mSystems.00129-17)

Figure S1

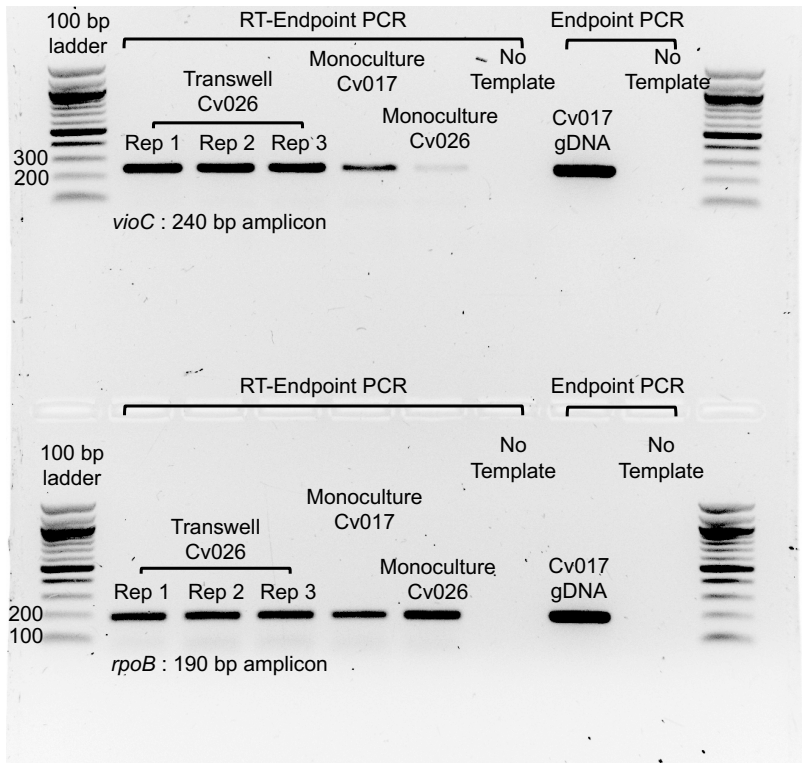

Supplement: FIG S1 [file sys006172151sf1.pdf]

**Figure S2**

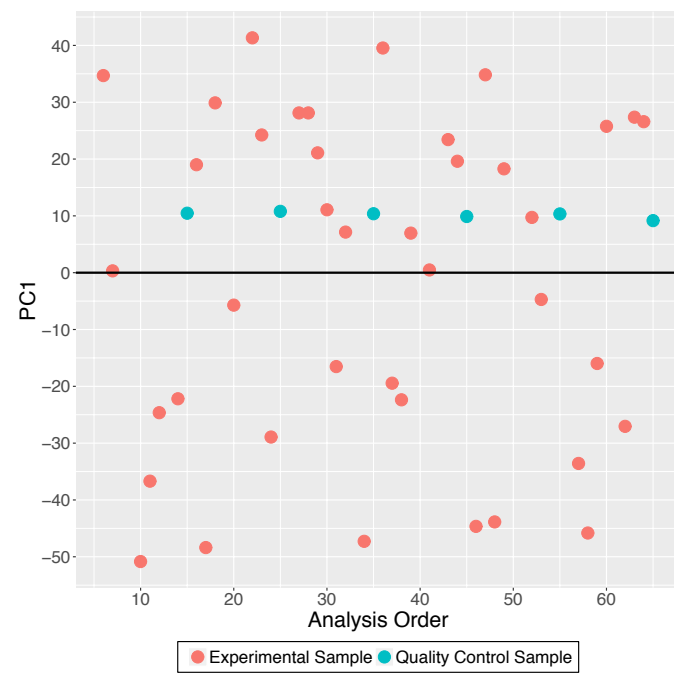

Supplement: FIG S2 [file sys006172151sf2.pdf]

Figure S3

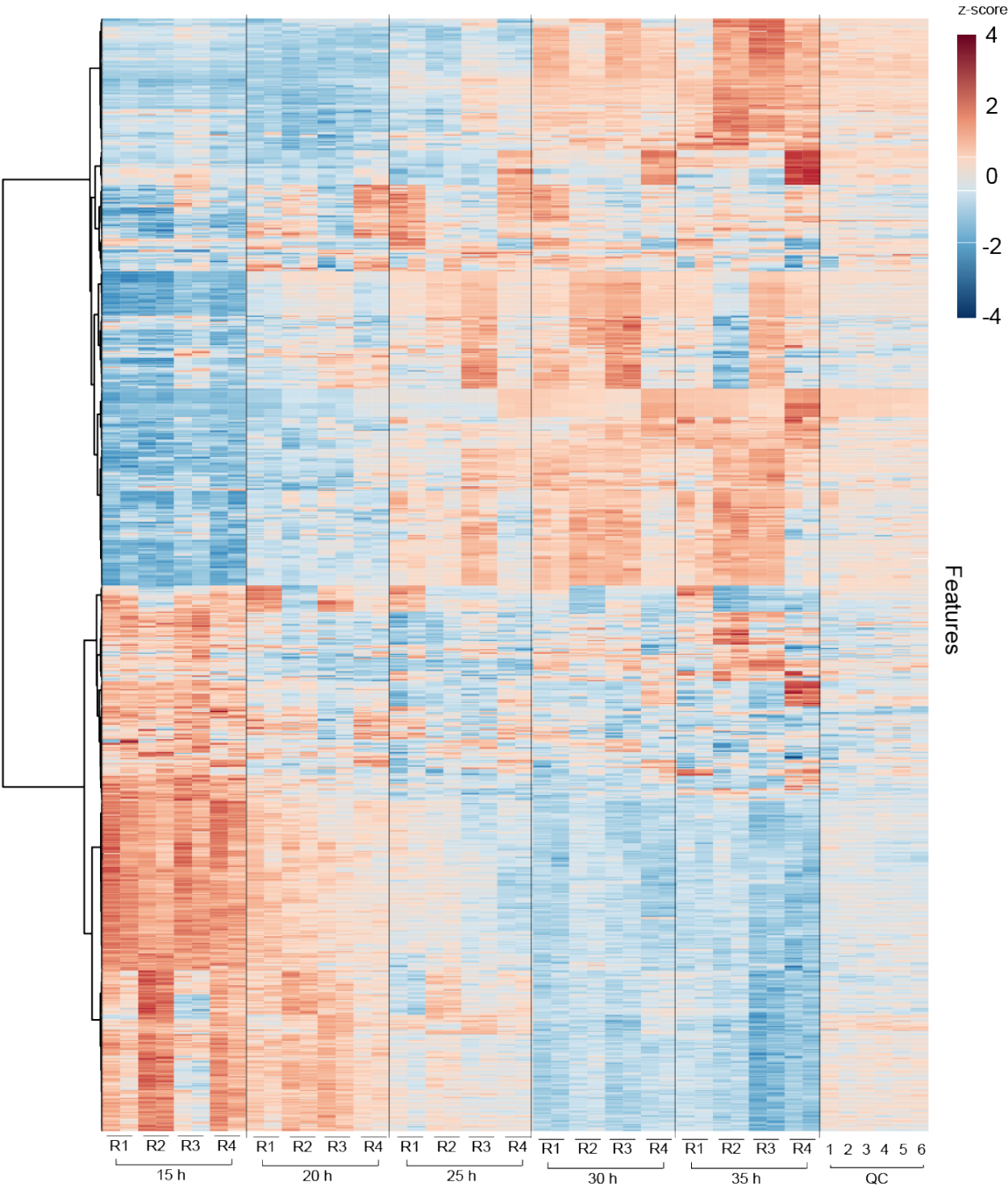

Supplement: FIG S3 [file sys006172151sf3.pdf]

**Figure S4**

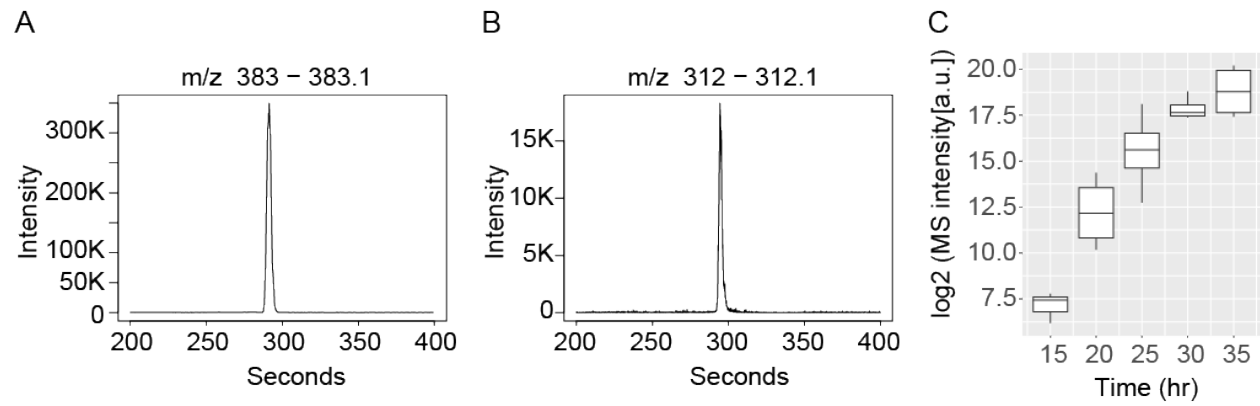

Supplement: FIG S4 [file sys006172151sf4.pdf]

Figure S5

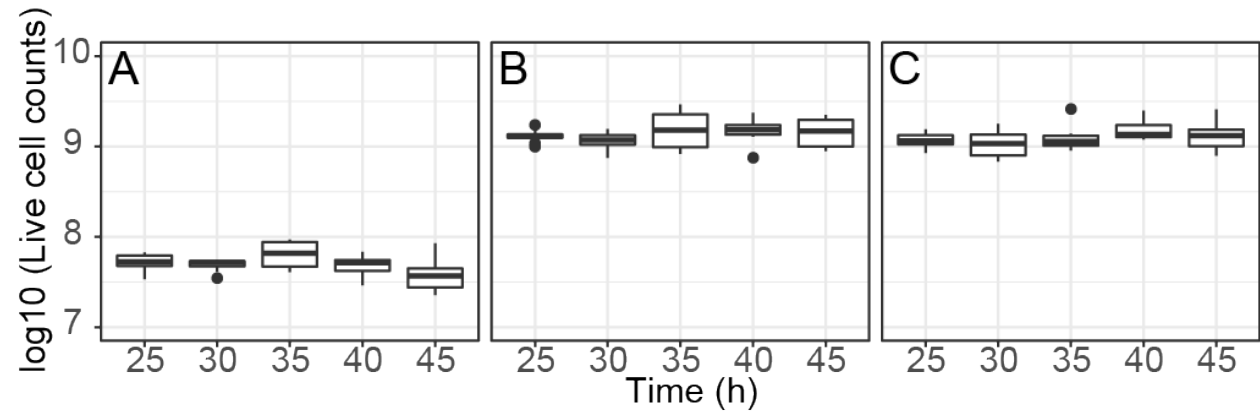

Supplement: FIG S5 [file sys006172151sf5.pdf]

Figure S6

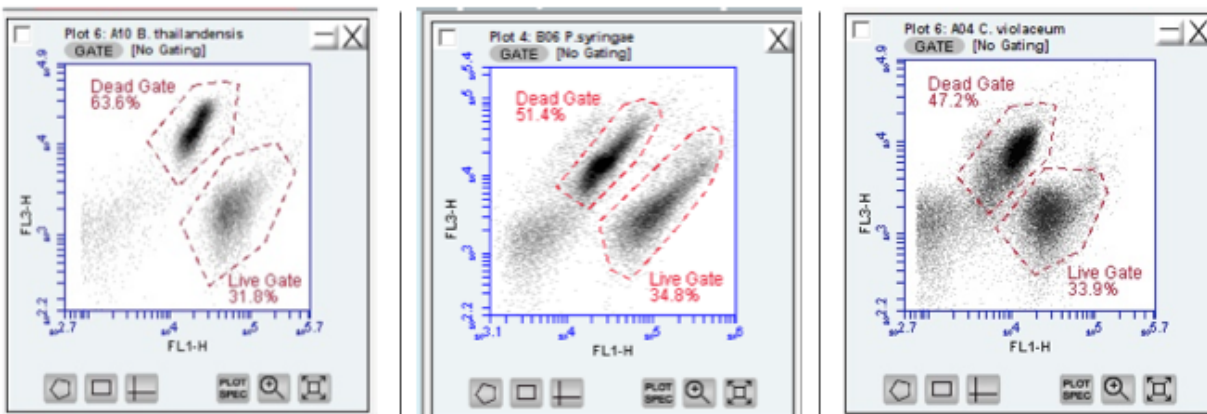

Supplement: FIG S6 [file sys006172151sf6.pdf]
