# Supplementary material for: A Synthetic Community System for Probing Microbial Interactions Driven by Exometabolites
Source: mSystems. 2017 Nov 14;2(6):e00129-17. doi: 10.1128/mSystems.00129-17 (PMC5686522; doi:10.1128/mSystems.00129-17)
Supplement: TABLE S1 [file sys006172151st7.pdf]

Supporting Tables

Table S1. **Fragments observed from MS/MS analysis of bactobolin**

MS/MS Fragments	Observed[a]	Calculated	PPM	Intensity (a.u.)
**1**	366.053	366.051	5.46	7E2
**2**	365.064	365.067	8.22	4.3E2
**3**	322.026	322.025	3.11	1.8E3
**4**	312.039	312.041	6.41	1.6E4
**5**	294.026	294.030	13.60	3.1E3
**6**	286.050	286.048	6.99	6.6E2
**7**	276.021	276.019	7.25	1.6E3

[a] MS/MS data were acquired under positive ionization mode. Bactobolin, (M+H)obs=383.075, (M+H)calc=383.077
