# Supplementary material for: A Synthetic Community System for Probing Microbial Interactions Driven by Exometabolites
Source: mSystems. 2017 Nov 14;2(6):e00129-17. doi: 10.1128/mSystems.00129-17 (PMC5686522; doi:10.1128/mSystems.00129-17)
Supplement: TABLE S2 [file sys006172151st8.pdf]

Table S2. ***C. violaceum*** **primer sets designed for reverse transcription and end-point PCR.**

Primer[a]	Primer (5' – 3')	Reference GenBank accession and location	Expected amplicon size (base pairs)	Citation
vioC_Cv Forward	GTCGATCTGGAAGGCAAGTC	LC000628.1:4401-5690	240	This study
vioC_Cv Reverse	CATGCCGAAGAAGTACAGCA			
rpoB_Cv Forward	GCTATGCCAAGCTGGACTTC	LKIW01000107.1:4687-8862	190	This study
rpoB_Cv Reverse	ATCTCGCCCATGTACACCTC			

[a] Primers were designed using the genome of *C. violaceum* Cv017(58).
